# Protective effect of hydroalcoholic extract of *Pistacia vera* against gentamicin-induced nephrotoxicity in rats

**DOI:** 10.1080/0886022X.2017.1326384

**Published:** 2017-05-30

**Authors:** Vahid Ehsani, Morteza Amirteimoury, Zahra Taghipour, Ali Shamsizadeh, Gholamreza Bazmandegan, Amir Rahnama, Fatemeh Khajehasani, Iman Fatemi

**Affiliations:** a Physiology-Pharmacology Research Center, Rafsanjan University of Medical Sciences, Rafsanjan, Iran;; b Department of Anatomy, School of Medicine, Rafsanjan University of Medical Sciences, Rafsanjan, Iran;; c Department of Physiology and Pharmacology, School of Medicine, Rafsanjan University of Medical Sciences, Rafsanjan, Iran;; d Department of Pathology, School of Medicine, Rafsanjan University of Medical Sciences, Rafsanjan, Iran;; e Department of Radiology, Rasoul-e-Akram Hospital, Iran University of Medical Sciences, Tehran, Iran

**Keywords:** *Pistacia vera*, gentamicin, nephrotoxicity, nephroprotective activity, rat

## Abstract

**Purpose:***Pistacia vera* is a plant of the family Anacardiaceae found in Central and West Asia. *P. vera* nut (Pistachio) possess multiple pharmacological effects such as antimicrobial, anti-hyperlipidemia, antioxidant and anti-inflammatory. This study is designed to evaluate the protective effect of the hydroalcoholic extract of pistachio on gentamicin-induced nephrotoxicity in rats.

**Methods:** Nephrotoxicity was induced in rats by intraperitoneal injection of gentamicin (100 mg/kg/day for 7 days). Hydroalcoholic extract of pistachio (10, 50 and 100 mg/kg/p.o) was administered for 7 days. The nephroprotective activity was evaluated by determining creatinine clearance, serum creatinine, urine volume, urine glucose and blood urea nitrogen (BUN) levels. The kidneys were processed for histopathological examinations and all specimens were examined for morphologic parameters involving tubular degeneration, tubular necrosis and tubule interstitial nephritis.

**Results:** Results showed a significant increase in the levels of serum creatinine, urine volume, urine glucose and BUN and decrease of creatinine clearance by gentamicin (GA) administration. Co-administration with pistachio extract showed reduction in the levels of serum creatinine, urine volume, urine glucose and BUN and increase of creatinine clearance in all doses but the most significant alteration was observed in doses of 100 mg/kg. Also, the nephroprotective effect of the GA was confirmed by the histological examination of the kidneys.

**Conclusion:** The study revealed the nephroprotective effect of the hydroalcoholic extract of pistachio. These findings suggest that pistachio treatment may attenuate renal dysfunction and structural damage through the reduction of oxidative stress and inflammation in the kidney.

## Introduction

Acute renal failure (ARF) refers to a sudden and usually reversible decrease in kidney function [1]. The pathogenesis of ARF is complex, however ischemia or toxins are known as the major underlying factors [2]. Nephrotoxic drugs such as cisplatin and aminoglycoside antibiotics are the main causes for nearly 20% of all ARF cases in intensive care units [3]. Aminoglycoside antibiotics – such as gentamicin (GM) – are used as effective agents against gram-negative bacteria infections [4]. About 30% of the patients, undergone GM treatment for more than seven days, display signs of nephrotoxicity [5]. The cellular mechanism/s of GM-induced nephrotoxicity is still poorly understood. Reactive oxygen species (ROS) have important role in pathological mechanisms of GM-induced ARF. Production and accumulation of ROS results in induction of apoptosis, tubular necrosis and increased infiltration of leukocyte [6]. This GM-induced ARF is clinically characterized by an increase in serum creatinine levels and urea nitrogen, a reduction in the glomerular filtration rate (GFR) and urine osmolality [7].

Several lines of evidence support the use of plant extracts for the prevention and attenuation of ARF [8]. *Pistacia vera* (*P. vera*) (family: Anacardiaceae) is native of arid zones of Central and West Asia and has commonly been used in traditional herbal medicine [9]. Pistachio (*P. vera* nut) have a valuable nutrient profile. It is a unique source of unsaturated fatty acids and numerous antioxidants, including α-tocopherol, β-carotene, lutein, selenium, flavonoids and phytoestrogens [10]. Previous studies have provided evidence suggesting various pharmacological properties for *P. vera* including antioxidant [11], anti-microbial [12], anti-nociceptive, anti-inflammatory [13] and hepatoprotective effect [14]. It has been shown that pistachio consumption has positive effects on serum lipid profile and CVD risk factors in hypercholesterolemic humans [15]. In a recent study in humans, it was observed that pistachio diet significantly improved oxidative status and decreased circulating inflammatory biomarkers [16].

Inflammation and ROS play significant roles in pathophysiology of ARF [17]; therefore, administration of compounds with antioxidant and anti-inflammatory properties induces ameliorative effects. The present study was designed to investigate the effect of hydroalcoholic extract of *P. vera* in a rat model of GM-induced ARF.

## Materials and methods

### Plant material and extraction method

Dried Pistachio from *Akbari* species with genetic code of *M30* were purchased from an herbal pharmacy in Rafsanjan, Iran. In order to prepare the required extract, dried and finely powdered fruits (100 g) were macerated in 1 L of methanol (80%) for 72 h to obtain the whole extract using the percolation method. Extract vehicle was evaporated in a rotary under low pressure. The extract was then frozen and stored at −20 °C. For administration, the frozen pistachio extract (PE) was freshly dissolved in dimethyl sulfoxide 10% (DMSO, Sigma-Aldrich, Germany).

### Animals

Forty-nine male Wistar rats (250–300 g) were obtained from the animal house of School of Medicine, Rafsanjan University of Medical Sciences, Rafsanjan, Iran. Animals were housed in polycarbonate cages under 24 ± 2 °C room temperature with a 12-h light/dark cycle and *ad libitum* access to food and water. All experiments were performed in accordance with the guidelines set by the ethical committee of Rafsanjan University of Medical Sciences and the European Communities Council Directive 24 November 1986 (86/609/EEC).

### Experimental design

Animals were divided into seven experimental groups as follows: group 1 (Control group) did not receive any solvent or drug during experiments and received a usual diet; group 2 (GM group) received 100 mg/kg of GA (Alborz Co, Tehran, Iran) intraperitoneally (i.p.) for 7 days; group 3 (DMSO group) received i.p. injections of 100 mg/kg of GA and DMSO 10% orally for 7 days; group 4 (D10 group) received i.p. injections of 100 mg/kg of GA and PE orally at the dose of 10 mg/kg for 7 days; group 5 (D50 group) received i.p. injections of 100 mg/kg of GA and PE orally at the dose of 50 mg/kg for 7 days; group 6 (D100 group) received i.p. injections of 100 mg/kg of GA and PE orally at the dose of 100 mg/kg for 7 days and group 7 (Extract 100 group) received PE orally at the dose of 100 mg/kg for 7 days to assess the possible toxic effects of PE.

### Sample collection and biochemical assays

On day 7 of experiment, 24-h urine samples were collected for measurement of urine volume and glucose concentration. Animals were sacrificed on day 8 of experiment, using ether anesthesia. Blood samples were taken by cardiac puncture and kept for 1 h at 4 °C. These were then centrifuged at 3000 rpm for 15 min to separate serum. The serum samples were stored for measurement of the blood urea nitrogen (BUN) and serum creatinine. The GFR (mL/24 h) was estimated by creatinine clearance. The serum and urine creatinine concentrations were determined by Jaffe’s method. BUN was measured colorimetrically using Autoanalyzer (Technicon RA-1000, London, England) and urea kit (Man Lab Company, Tehran, Iran). Urinary glucose concentration was measured by the enzymatic assay (glucose oxidase) and protein concentration was assessed *via* turbidimetric method.

### Histopathological examination

Both kidneys were immediately removed and fixed in 10% neutral buffered formalin for histopathological examinations. The kidney tissues were dissected out, washed by normal saline solution (0.9%) and then fixed in 10% formalin solution for 48 h. The kidneys were processed for dehydration using absolute ethanol, cleaned in xylene, embedded in paraffin and sectioned for histopathological evaluations. The prepared sections were stained with haematoxylin and eosin and were then visually observed under light microscope. All specimens were examined for three morphologic parameters, including tubular degeneration (TD), tubular necrosis (TN) and tubule interstitial nephritis (TIN) on a semiquantitative score from 0 to 4 [1]. The score of zero was assigned to the normal tissue with no damage.

### Statistical analyses

Statistical analyses were performed by Excel 2007 (Microsoft Corporation, Seattle, WA) and SPSS 18 software (SPSS Inc, Chicago, IL). Results are presented as mean ± standard error of the mean (SEM). Differences between groups were determined using ANOVA followed by the Tukey *post hoc* test. Values of *p* < .05 were considered significant.

## Results

### Biochemical assays

**Figure 1.**
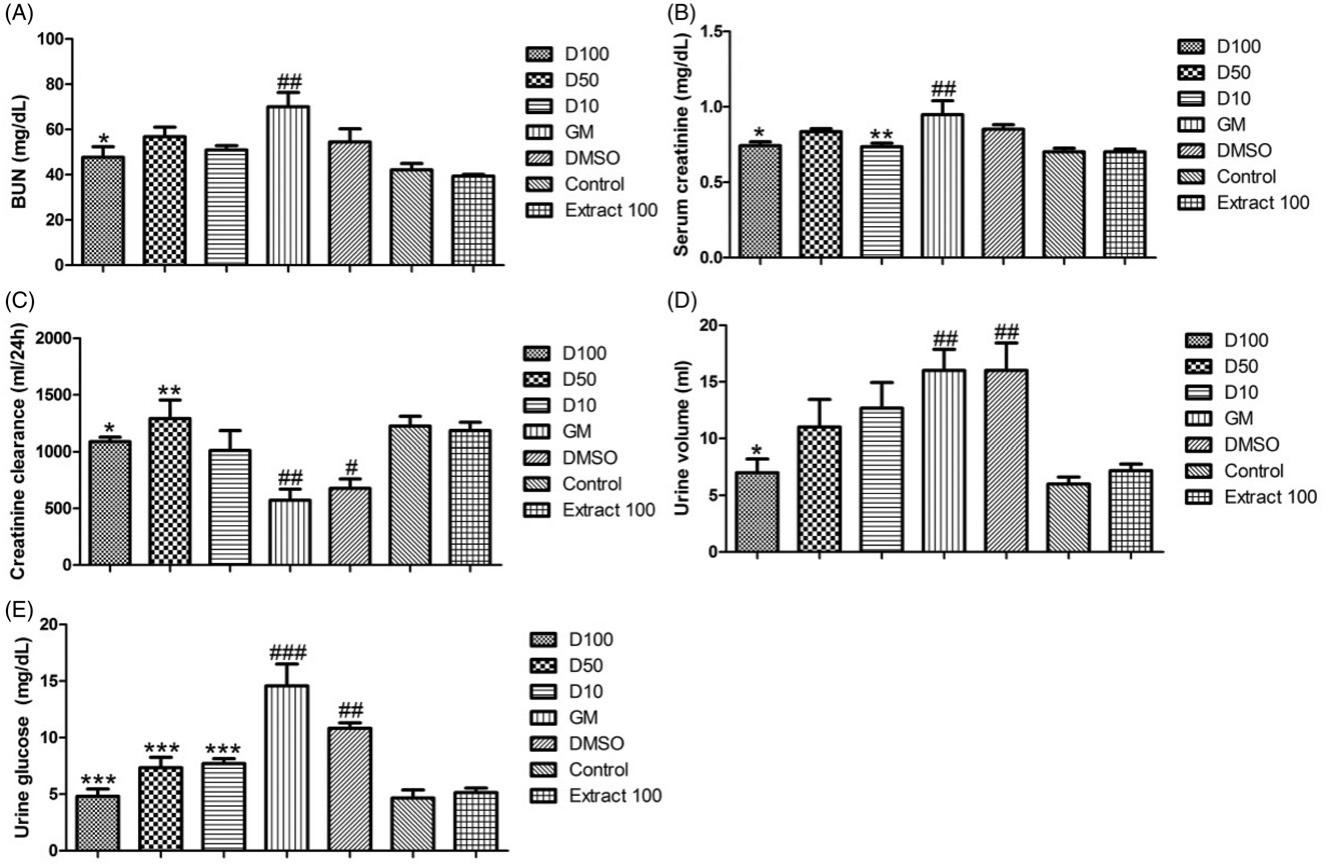
Effect of PE (10, 50 and 100 mg/kg) on concentrations of BUN (A), serum creatinine (B), creatinine clearance (C), urine volume (D) and urine glucose (E) in rats with GM-induced ARF. Data are expressed as mean ± SEM and analyzed by one-way ANOVA followed by *post hoc* Tukey tests. *p < .05, **p < .01, ***p < .001 as compared with the GM group and #*p* < .05, ##*p* < .01, ###*p* < .001 as compared with the control group.

PE induced a significant nephroprotective effect and most GM-induced renal alterations were not observed following co-administration of PE + GM (Figure 1). In animals treated with 100 mg/kg PE, the concentrations of BUN (*p* < .05), serum creatinine (*p* < .05), urine volume (*p* < .05) and urine glucose (*p* < .001) were significantly decreased compared to the GM group, however creatinine clearance (*p* < .05) showed a significant increase compared to the GM group. In rats treated with 50 mg/kg PE, the concentrations of urine glucose (*p* < .001) were significantly decreased compared to the GM group, but creatinine clearance (*p* < .01) showed a significant increase compared to the GM group. In groups treated with 10 mg/kg PE, the concentrations of serum creatinine (*p* < .01) and urine glucose (*p* < .001) were significantly decreased compared to the GM group. In addition, administration of 100 mg/kg PE did not elicit any clinical sign of toxicity, renal dysfunction and mortality for a period of 7 days.

### Histopathology

**Figure 2.**
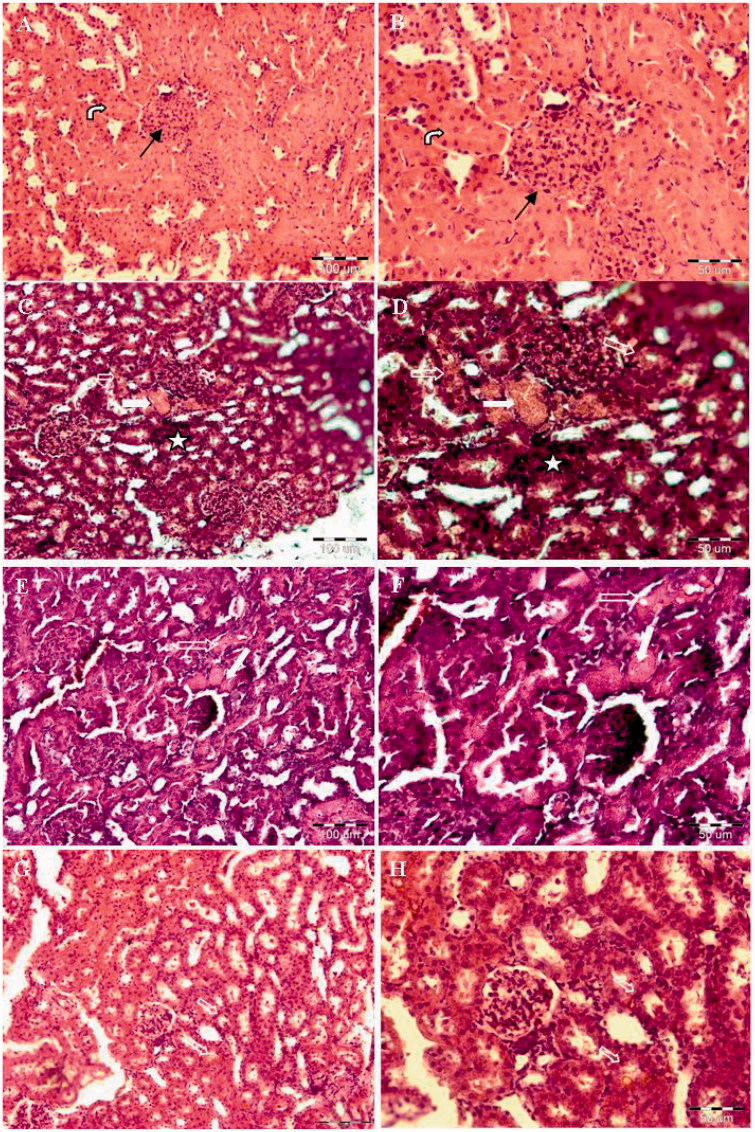
Effect of PE on the morphology of the rat kidneys with GM-induced ARF. Control group: healthy kidney structure was seen. The glomerulus (arrow) and tubules (bent arrow) are normal (A: ×20 and B: ×40). GM group: kidney is severely damaged. Acute tubular necrosis (filled arrow) and extensive tubular degeneration (thick arrow) were seen. Sever leukocyte infiltrations in intertubular area were also found (star) (C: ×20 and D: ×40). D50: minimal tubular necrosis and tubular degeneration (thick arrow) were observed. Slight leukocyte infiltrations in intertubular area are still seen (E: ×20 and F: ×40). D100: showed dramatic improvement in the morphologic appearance. Tubular degeneration (thick arrow) and leukocyte infiltrations have been recovered (G: ×20 and H; ×40).

**Figure 3.**
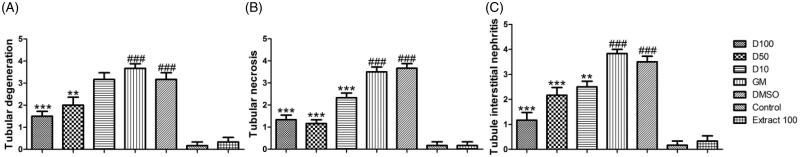
Effect of PE (10, 50 and 100 mg/kg) on morphologic parameters, including tubular degeneration (A), tubular necrosis (B) and tubule interstitial nephritis (C) in rats with GM-induced ARF. Pathological examination performed by semiquantitatively scored from 0 to 4. Data are expressed as mean ± SEM and analyzed by one-way ANOVA followed by *post hoc* Tukey tests. **p < .01, ***p < .001 as compared with the GM group and ###*p* < .001 as compared with the control group.

In order to evaluate the effect of PE on the histological changes in the kidney, H&E staining was performed (Figure 2). Histopathological scores of TD, TN and TIN in all experimental groups were graded (Figure 3). Sections from kidney tissues of GM treated rats showed massive TD, TN and TIN (Figure 3), while co-administration of PE + GM reduced these parameters in renal tissues compared to the GM group in a dose–response manner. Inanition, administration of 100 mg/kg PE did not cause any detectable alteration in the renal structure of the normal rat.

## Discussion

In the present study, the effect of PE on GM-induced ARF was investigated in rats. Results indicated that intraperitoneal administration of GM (100 mg/kg) results in significant nephrotoxicity as evidenced by increase in serum creatinine, urine volume, urine glucose and BUN levels as well as sever TD, TN and TIN which was consistent with previous reports [5,18,19]. Treatment with PE increased the GM-induced attenuation of creatinine clearance and decreased the GM-induced enhancement of serum creatinine, urine volume, urine glucose and BUN levels. Moreover, we found that administration of PE for 7 days significantly decreased the TD, TN and TIN scores. We also demonstrated that administration of PE (100 mg/kg) in normal rats for 7 days did not alter the kidney morphologically and functionally. The results of the present study, for the first time, indicated that oral administration of PE had a significant and to some extent dose-dependent protective effect on GA-induced nephrotoxicity in rats.

Aminoglycosides are commonly used against gram-negative pathogens [20]. In recent years, the consumption of these drugs has been reduced due to the induction of nephrotoxicity and ototoxicity. Among the aminoglycoside antibiotics GA has been used worldwide due to its availability, effectiveness and cost especially in developing countries [21].

Currently, it is well established that the most important mechanism of GM-induced nephrotoxicity is overproduction of ROS like hydroxide and hydrogen peroxide causing renal cell damage [22]. This overproduction of ROS is associated with depletion of renal antioxidant enzymes [23]. ROS damage the protein molecules and alter the cellular membrane integrity *via* lipid peroxidation processes which in turn results in morphological and functional changes [24]. The nephroprotective effects of antioxidant compounds have been reported. Sahu et al. have shown that naringin attenuates renal dysfunction and GM-induced structural damage *via* reducing the oxidative stress. They suggested that antioxidative effect of naringin reduces the inflammation and apoptosis in the kidney. In another study, Jafarey et al. suggested that the antioxidative effect of calcium dobesilate mitigates the nephrotoxicity caused by GA [25]. In addition, pistachio has been ranked among the 50 antioxidant-rich foods [26]. Pistachio have some component with high antioxidant activity such as polyphenols, tocopherols, lutein, phytosterols, vitamin B6, gallic acid and carotenoids [27]. Kocyigit et al. have shown that the consumption of pistachio significantly decreases oxidative stress and improves plasma lipid profile in healthy volunteers [28]. Also, Shahraki et al. have reported the hepatoprotective effect of PE against ROS formation and lipid peroxidation. They have demonstrated that methanolic extract of pistachio has ROS and carbonyl scavenging activity and inhibits lipid peroxidation process [9]. Moreover, in a recent study in humans Gentile et al. showed that pistachio significantly improves the oxidative status and reduces the circulating inflammatory biomarkers in inflammatory bowel diseases [16]. These observations support the hypothesis that the nephroprotective effect of PE might be attributed to direct attenuation of ROS (antioxidant activity) and reinforcement of the antioxidant system.

Our results showed that the concurrent administration of PE significantly decreases the histopathological scores compared to the GM-treated group. Accumulation of GM in the renal tubules is another mechanism underlying GM-induced nephrotoxicity [6]. This accumulation could result in tubular degeneration and necrosis as well as stimulating inflammatory events and promoting the migration of monocytes and macrophages at the site of renal injury [29]. Attenuating the inflammatory processes and leukocytes recruitment have been shown to improve the GFR and renal functional parameters [22]. It has been reported that treatment with GA increases NF-κB activation, cyclooxygenase-2 expression [2] and levels of pro-inflammatory cytokines such as TNF-α and IL-6 [22]. On the other hand, the anti-inflammatory effects of *P. vera* have been previously demonstrated. Gentile et al. have shown that pistachio decreases cyclooxygenase-2 expression, IL-6 and IL-8 release and NF- κB activation [16]. Accordingly, *P. vera* may possibly improve histopathological scores and decrease leukocytes infiltration through the suppression of inflammatory process.

Treatment with several herbal extracts has been extensively studied and shown to be useful for either the prevention or amelioration of drug-induced nephrotoxicity [30–33]. Boroushaki and Sadeghnia demonstrated the protective effect of Safranal (the main constituents of saffron extract) against GM-induced nephrotoxicity in rat [34]. In another study, Kang et al. suggested that *Houttuynia cordata* induces renoprotection by reduction of oxidative stress in GM-induced ARF [35]. Moreover, Nasri et al. reported the protective effect of Garlic against GM-induced nephrotoxicity [36]. The renoprotective activities of these plants have been attributed to the antioxidant properties of the plants. Other antioxidants have also revealed renoprotection against nephrotoxic agents [30,37–39]. Hence, the renoprotective property of *P. vera*, at least in part, might be related to its antioxidant activity. There are a lot of other plants with antioxidant activity [40–42], and, if we accept this conclusion, they should also have renoprotective activity, which worth examining.

## Conclusion

The data gathered in the present study suggest that the methanolic extract of *P. vera* possesses potential protective activity against GM-induced ARF. We also found that treatment with PE increases creatinine clearance and attenuates the serum creatinine, urine volume, urine glucose, BUN levels and histopathological scores. This may stand to reason that PE has antioxidant and anti-inflammatory effects. However, further investigations are required to unveil the precise underlying cellular mechanisms.
